# Barriers to utilization of childbirth services of a rural birthing center in Nepal: A qualitative study

**DOI:** 10.1371/journal.pone.0177602

**Published:** 2017-05-11

**Authors:** Resham Bahadur Khatri, Tara Prasad Dangi, Rupesh Gautam, Khadka Narayan Shrestha, Caroline S. E. Homer

**Affiliations:** 1 Center for Research and Development, Kathmandu, Nepal; 2 Ministry of Home Affairs, Government of Nepal, Kathmandu, Nepal; 3 Department of Public Health, Aarhus University, Aarhus, Denmark; 4 Department of Sociology and Anthropology, Tribhuvan University, Kathmandu, Nepal; 5 Faculty of Health, University of Technology Sydney, New South Wales, Australia; National Academy of Medical Sciences, NEPAL

## Abstract

**Background:**

Maternal mortality and morbidity are public health problems in Nepal. In rural communities, many women give birth at home without the support of a skilled birth attendant, despite the existence of rural birthing centers. The aim of this study was to explore the barriers and provide pragmatic recommendations for better service delivery and use of rural birthing centers.

**Methods:**

We conducted 26 in-depth interviews with service users and providers, and three focus group discussions with community key informants in a rural community of Rukum district. We used the Adithya Cattamanchi logic model as a guiding framework for data analysis.

**Results:**

Irregular and poor quality services, inadequate human and capital resources, and poor governance were health system challenges which prevented service delivery. Contextual barriers including difficult geography, poor birth preparedness practices, harmful culture practices and traditions and low level of trust were also found to contribute to underutilization of the birthing center.

**Conclusion:**

The rural birthing center was not providing quality services when women were in need, which meant women did not use the available services properly because of systematic and contextual barriers. Approaches such as awareness-raising activities, local resource mobilization, ensuring access to skilled providers and equipment and other long-term infrastructure development works could improve the quality and utilization of childbirth services in the rural birthing center. This has resonance for other centers in Nepal and similar countries.

## Introduction

The majority of maternal deaths globally are caused by haemorrhage, sepsis, unsafe abortion, obstructed labor and hypertensive diseases of pregnancy, and many would be preventable with access to adequate, appropriate health services [1]. At least 300,000 women around the world still die annually due to pregnancy-related complications with 95% of these deaths taking place in the low and middle-income countries [2]. Furthermore, maternal complications, such as obstetric fistula, can lead to long-term disability and cause women to be ostracized from their families and communities [3]. Maternal health deserves attention as it is intricately linked with socio-cultural context of society [4, 5]. In many countries, socio-environmental conditions such as gender biases, combined with poverty, stressful work environments and a poor quality of life mean women have inadequate nutrition, experience early marriages and repeated pregnancies thereby exacerbating the risk of morbidity and mortality [6].

In Nepal, marrying girls at a young age is usual. One study reported that one-third (32%) of Nepali girls were married before the age of 16 years, and 78% women were married by the age of 20 years (the legal age of marriage) [7]. There is also a relief associated with marrying daughters amongst parents, especially in the most rural areas and southern plains where the poverty rates are higher [8]. These practices mean that women become pregnant (56%)in adolescence [9]. Early marriage and childbearing put women at further risk of maternal morbidity and mortality. Early childbearing usually means young women drop out of school which leads to poor education, further limiting future employment prospects. This creates a vicious cycle of early marriage, adolescent pregnancy, and unemployment, ultimately leading into an inter-generational poverty trap.

Nepal is one of the countries in the world with a high maternal mortality ratio [10]. The progress on maternal mortality ratio reduction is slow. Between 1996 and2006, the maternal mortality ratio reduced from 539 to 281 per 100,000 live births [11]. Inequality among the disadvantaged groups is another challenge. The Nepal Maternal Morbidity and Mortality Survey 2008/09 reported that 41% of maternal deaths occurred at health facilities, 40% at home, and 14% on the way to health institutions [11, 12]. According to Nepal’s Sustainable Development Goals (SDG) implementation plan, Nepal has set targets of reducing MMR to 70 per 100,000 live births and achieving 90% coverage of four antenatal care (4ANC) visits, institutional births by skilled birth attendants (SBA) and instituting postnatal care visits by 2030 [13]. It will be challenging to reduce deaths and achieve routine care to meet the SDGs unless focused or contextual policies and strategies are implemented that effectively target socio-cultural practices and widespread disparities.

Nepal has 125 ethnicities, and 123 languages within a small geographical boundary [14] and these diverse groups have various cultural beliefs regarding pregnancy and delivery care [15]. For instance, in many communities, pregnancy is considered culturally as a matter of fate and a natural process that does not warrant extra attention [16, 17]. Shyness, avoiding touch from males during labor and birth, and the dominance of mother-in-law in decision making(even regarding where to give birth) are common. These issues can lead to delays in deciding to seek care[18]. In some areas, deaths during childbirth are believed to be inflicted by evil supernatural forces or spirits and thus seeking help from traditional healers before consulting skilled obstetric or midwifery care is common [19]. Delays in reaching health facilities due to difficulty in transportation are also commonly reported [20]. When the women do reach health facilities, health workers are absent, unavailable or in some cases, even disrespectful to women [21].

In rural areas of Nepal, about 65% of births still take place at home without the assistance of SBAs [22]. Substantial equity gaps are observed between wealthy and poor women [10]. Between 2001 and 2011, childbirths assisted by SBAs increased from 4% to 11% among the lowest wealth quintile but increased from 45% to 82% among the highest wealth quintile [10]. Keeping the access and equity issues in mind, the Government of Nepal since the late 1990s has implemented Safe Motherhood as a national flagship program [22]. Under this program; the Maternity Incentive Scheme (in Nepali-Aama Suraksha Karyakram) was implemented in 2005 [23]. The aim of Maternity Incentive Scheme is to encourage women to have facility-based births by addressing demand side financial barriers[23]. The Maternity Incentive Scheme includes the provisions for incentives (≈5, 10 and 15 USD in Plain, Hills and Mountain regions respectively) to women who give birth at the facilities including the birthing centers. Four years later in 2009, an extra incentive (≈4 USD) was added (in addition to facility-based births) for women who complete at least four ANC visits as per the ANC protocol (at the 4^th^,6^th^,8^th^ and 9^th^months of pregnancy) [24].

In Nepal, birthing centers are the lowest health units where facility-based births are available. The birthing centers house at least one Auxiliary Nurse Midwife who is trained on SBA package [25]. The SBA package is a two-month long, focused training on emergency obstetric and neonatal care including antibiotics, anticonvulsants, and uterotonics, clean cord care and neonatal resuscitation. Auxiliary Nurse Midwives have 18-month midwifery training after completing 10^th^ grade in high school. They are accredited as SBAs after additional specific training that enables them to provide childbirth services. Despite this requirement, Nepal has an acute shortage of SBAs. In principle, a birthing center must have an SBA-trained Auxiliary Nurse Midwife, but many birthing centers are being run by Auxiliary Nurse Midwives who do not have the additional SBA training. Around 1500 birthing centers are supposed to provide childbirth services in the rural parts of the country. However, only10% of the total births each year take place in those centers [26]. Many women prefer giving birth either at home or, if available and accessible, going directly to the district or referral hospitals. It means that the rural birthing centers are often bypassed or underutilized [27]. Taking this into account and to increase the service utilization, a program known as “Warm Bags (*Nyano Jhola*)” was implemented in 2011. This program involves the distribution of warm clothes for mothers and newborns in return for giving birth at the birthing centers. However, the rate of institutional births in those facilities has remained stagnant over the four years since the implementation of Warm Bags program [28].

A few studies have explored the utilization, preferences, and experiences towards facility-based births. However, these studies were inadequate in documenting the reasons for underutilization of rural birthing centers [29–31]. Therefore, the aim of this study was to explore the barriers and provide pragmatic recommendations for better service delivery and use of rural birthing centers.

## Methods

### Study setting

The study site is one of the remotest Village Development Committees in Rukum district of the mid-western region, Nepal. This site is located more than 40 kilometers north of the district headquarters. In Nepal, a Village Development Committee is the smallest administrative unit and is further divided into nine wards for service delivery and development purposes. The study site covers more than 200 square kilometers [32].

The site has a population of 7,000 (1400 households). The dominant ethnicity is Brahmin/Chhetri (66%) followed by Dalits (33%), and most people speak Nepali. In the 2011 national census, the literacy rate in the district was lower than the national average (male 71% and female 54%) [14]. The literacy rate of the study site may be lower due to its remoteness. The economic status of households has divided into ultra-poor (19%), poor (36%), middle class (34%) and the remaining as privileged (11%) [33]. Usually, the poor and those with Dalit ethnicity were found to live in the remote parts of the study site.The major occupation is agriculture (77%) followed by work as a porter, (20%), business (6%) and the service sector (3%)[33]. Roads are not connected to the study site.The major means of transportation for supplies are donkeys and mules. It takes eight hours to reach the nearest motorway and two days to the referral center (district hospital). During an emergency, stretcher or human-made carrier (called *doko* in Nepali) are the only options to carry the referred individuals.

The study site has one birthing center staffed with four health workers. Every month, health workers conduct three immunization clinics and two primary health care outreach clinics to provide maternal and child health services in hard to reach wards of the Village Development Committee. There is also a Female Community Health Volunteer in each ward who provides promotive and preventive health services to women and children under five years. Child marriage is common in this village with more than 180 married couples aged below 14 years[32]. Given the population and fertility rate, it is estimated that the study village would expect around 154 pregnancies annually. The record of birthing center register showed that 55 women (35% of expected pregnancies) gave birth at the birthing center one year before this study [34].

### Study design

This was a qualitative study using in-depth interviews with service users and health service providers and focus group discussion sessions with the community stakeholders. The service users were women (or their husbands) who were living in the study area and had given birth either at home or the birthing center one year prior to the data collection. The service providers were health workers who had paramedical or midwifery training and were working at the birthing center or running a pharmacy in the study site. The community stakeholders were social workers, leaders, teachers or civil servants who were involved formally or informally in social service and development at the study site. The study participants were selected purposively using the process described below.

From the maternity register, we developed a list of women who had given birth at the birthing center. The Female Community Health Volunteers also had a list of women who were pregnant each year. We made home visits with the Female Community Health Volunteers and identified at least two potential women from each ward: one woman who had given birth at a birthing center and another who had given birth at home. From one ward, we also identified one more interviewee whose wife had died due to maternal complications. All health workers working at the birthing center, including one pharmacy worker, were invited to participate in the interviews.

Participants in the three focus groups included: 1) the health mothers’ group which is a formal group in each ward that includes pregnant women, mothers with under-five children, and the Female Community Health Volunteer from that ward. They usually meet every month and discuss their health-promoting activities. 2)The members of health facility operation management committee included one Female Community Health Volunteer, teachers, public servants local leaders, and the person in-charge of the birthing center (who is also member secretary of the committee). 3) Key political leaders,civil servants (local registrar) and chairman of Ward citizen forum [Table 1].

**Table 1:** Summary of methodology, participants, and contents of question guides.

Data collection methods	Participant details	Contents of question guides
**In-depth interviews**	21 service users: 20 women (10 from each group: childbirth at home and childbirth at a birthing center, one year prior to interviews) and a man whose wife had died due to childbirth complications.	Barriers to the use of birthing center for childbirth services including local context; Contributing factors to give birth at home; Awareness on importance of facility-based births, maternity incentive and birth preparedness plan; Perceived quality of services at the birthing center. Factors affecting provision of standard quality of facility-based births including safe working environment, equipment, and human resources; Barriers to service utilization
**In-depth interviews**	Five health service providers: one in-charge, two community health workers, one SBA and one pharmacy worker.	Barriers towards the use of facility-based births Perceived quality of services of the birthing center Factors affecting provision of standard quality of facility-based births including safe working environment, equipment, and human resources; Barriers to the utilization of facility-based birth services.
**Focus group discussions**	Three groups of community key informants: Focus group 1:(Health Mothers’ Group; 11 members): five pregnant women, five women with children aged under-five years, and one Female Community Health Volunteer Focus group 2: (Health facility operation management committee; nine members): The in-charge of the birthing center, three local social workers/teachers, one Female Community Health Volunteer and three community people. Focus group 3 (political leaders and civil servants): three local leaders- one each from the main political parties, chairpersons of ward citizen forum (ward number 3, 4 and 9) and the local registrar.	Local traditions, beliefs and cultural practices on childbirth; Perceived barriers to facility-based birth services from users’ and providers’ perspectives Barrier to quality of care and service delivery, Equipment and human resources

### Data collection

After reviewing previous studies on barriers to utilization of services of SBAs and based on our professional experiences, the first and second authors developed a series of guiding questions for the interviews and focus group discussions [20, 27, 35, 36]. The entry point for the generation of question guides was service utilization or service delivery, whether it was systematic or influenced by local context. These included barriers, logistics, human resources, service availability, quality of services, reasons for home birth, local support, challenges of service provision and local trust, and socio-cultural practices [Table 1]. The question guides were pre-tested in a nearby village, and some modifications (especially on local words) were made. The question guides were also somewhat flexible, where interviewers could probe the interviewees when needed.

One research assistant was recruited and oriented for the data collection and note taking procedures. The second author and the research assistant were from the study district, so they were familiar with local context but were not known to participants. The second author and research assistant visited the homes of selected participants and explained the purpose of their visits. After receiving consent, the second author undertook the face-to-face in-depth interviews in the Nepali language. Interviews were recorded on audio tape, and the research assistant also took notes in a diary. Confusing and important questions were repeated and probed in-depth to explore their experiences of childbirth practices. Data saturation was reached by the 26^th^ interview with no new factors being identified.

The focus group discussion with the Health Mothers’ Group was conducted in one ward of the study site. After completion of data collection at household and community levels, the interviews with health service providers, two focus group discussions (with management committee and local leaders) were conducted at the birthing center. Each interview and focus group discussion took an average of one to two hours. The data collection time was during the post-monsoon season (October to November 2014) which meant people had more time to participate. All our invited participants responded and agreed to participate.

### Data analysis

We adopted the logic model of Adithya Cattamanchi [37] to analyse the data. This model groups barriers into two broad categories: health system and contextual issues. Health-system barriers are institutional level factors such as drugs and equipment, human resources, service implementation and the coordination of services (supply and management). Contextual barriers are economic, geographic and socio-cultural factors that are linked to the uptake of services.

After completion of data collection, the first and second authors (RBK and TPD) analyzed the data. The first stage was transcription, in which three data sheets were developed for each category of participants. Initially, the audio records of each interview were listened to again. Then notes were taken about the important ideas of each audio recording and these were discussed and clarified by the two authors. The notes were then transcribed into Nepali. The transcriptions were again cross-referenced with the audio records and notes to check for content and accuracy. In the second stage, important ideas from each transcription were coded. The coded contents were translated into English, and this translation was reviewed by a third author (RG) for its accuracy and corrections. In the third stage, each interview response was sorted into broader category (health system or contextual). Within the broader category, after reviewing contents, similar ideas were organized into smaller sub-themes from each respondent’s category. If 25–30% of the participants (at least six women or two service providers or participants from focus group session) reported it, that sub-theme was taken as a barrier for that respondent category. Important verbatim quotes were also included in each barrier. Finally, if the same barriers were reported by at least two categories of participants, it was considered for final presentation.

### Research ethical approval

This study received ethical approval from the Institutional Review Committee (IRC) of the Department of Sociology and Anthropology, Tribhuvan University. The permission was also provided by the District Health Office. Before data collection, the purpose of research was described. Also, the participants were assured on the confidentially. Then the IRC-approved verbal informed consent form was read and participants were asked whether they agreed to participate in the research process. Further, it was explained that participants could terminate the interview at any time. After completing this procedure, if participants were still willing to take part in research, the data collection commenced.

## Results

### Socio-demographic features of participants

A total of 53 people participated in this study. Most (n = 31) were aged 20–40 years and were women (n = 33). Seventeen participants were illiterate, had poor socio-economic status (n = 28), and14 were Dalits (disadvantaged ethnicity) [Table 2].

**Table 2:** Summary of socio-demographic features of participants.

Variables	Categories	Service users (N = 21)	Service providers (N = 5)	Community key informants (N = 27)	Total
Age (years)	Below 20	7	0	5	12
	20–40	13	4	14	31
	40 and above	1	1	8	10
Sex	Male	1	3	16	20
	Female	20	2	11	33
Education (years of schooling)	Illiterate (0 years)	9	0	8	17
	Primary (1–8 years)	8	0	10	18
	Secondary (9–12 years)	4	5	9	18
Occupation	Housewife	15	0	2	17
	Agriculture	5	0	10	15
	Jobs	0	5	6	11
	Social services	0	0	6	6
	Business	1		3	4
Socioeconomic status	Poor	13	0	15	28
	Middle class	8	5	12	25
Ethnicity	Brahmin/Chhetri	12	5	18	35
	Dalits	8	0	6	14
	Indigenous	1	0	3	4

### Barriers

Five health system and four contextual barriers were identified. The most reported barriers were difficult geography, poor quality, and unavailability of 24-hour services, inadequate equipment and harmful local traditions [Table 3]. Almost all participants agreed that geographical inaccessibility was a major barrier to utilization of the birthing center.

**Table 3:** Summary of barriers to the utilization of a rural birthing center.

Broader barriers	Specific barriers	Service users	Service providers	Community key informants
**Health system barriers**	Unavailability of 24-hour services	+++	+	++
	Inadequate equipment	+	++	++
	Inadequate human resources		++	+
	Poor quality services	+++	+	++
	Poor governance (coordination and support)	+	++	
**Contextual barriers**	Difficult geography	+++	+++	+++
	Harmful cultural practices	+	++	+
	Low level of trust	++		++
	Poor birth preparedness plan	++	+	

“+” = 25–30% of the participants agreed, “++” = 50–60% of the participants agreed, and “+++” = 75–90% of the participants agreed.

### Health system factors

#### Unavailability of 24-hour services

When the women reached the birthing center in labor, the Skilled Birth Attendant (SBA) was often not there and often took half an hour or more to arrive from her residence. Although the national maternity care guideline states that it is mandatory to open the birthing center 24 hours a day, seven days a week, the SBA was often only present during regular office days (Sunday to Friday) and normal working hours (10 am to 5 pm). Occasionally, upon arrival, the companions with the women had to search for the SBA. One service user shared her experience linked with visiting the birthing center:

“When I visited there for antenatal care for the first time, the center was closed. I visited again next time, but the nurse was absent in that instance. After that, I did not make it a point of visiting there again for antenatal care, and I gave birth at home”

*[A woman who gave birth at home, 23 years old]*.

There was no office quarter or staff accommodation at the birthing center and neither was there a shop or cafeteria/canteen for having a meal in the immediate surroundings of the health facility. Therefore, the SBA had to live in the neighbouring village which was at least half an hour away from the birthing center. Sometimes, she would be undertaking her usual chores, such as cooking or doing the laundry, when she was suddenly called to attend births. This also contributed to further delays. As the SBA was not from the local community she left the area to visit her family or take holiday leave for important reasons such as festivals. This meant that she was not always available for pregnant women or those in labor. Furthermore, the SBA was working on her own with no certainty of when and what time of the day there would be a call for her services. She explained her situation below:

“It is hard to provide service at birthing center without any security and support; even we do not know when women come for childbirth”

*[SBA, 28 years]*.

Most of the community key informants reported that the birthing center opened for minimal hours and the staff provided services for an even shorter duration than the normal office hours. A local leader explained:

“The health facility is open only from 10:30 am to 2:00 pm. Why then would people come to receive services here within the limited hours? What is to be done if the labor pain starts beyond these hours?”

[Local leader, 35 years]

#### Inadequate human resources

The birthing center should have at least one SBA, and the institution should provide 24*7 childbirth services. Some birthing centers provide services with a permanent Auxiliary Nurse Midwife or temporarily recruited a local Auxiliary Nurse Midwife who did not have the additional SBA training. This latter option sometimes created problems. In an instance, a temporary provider’s contract ended after six months of recruitment and, because of the administrative procedures, it took another six months to recruit her again for the following year. In those six months, the health facility could not make use of her service despite her availability and readiness for the job.

The birthing center had only one SBA. Therefore, it was impossible for one SBA to provide 24-hourservices. The in-charge of the birthing center explained:

“We have only one SBA working here, and the birth service is disturbed when she becomes sick or goes on a leave or for training. We are helpless since we have no authority and fund for hiring an SBA when needed. The district health office has its own procedure for the recruitment of SBA, but it is very lengthy”

[In-charge of the birthing center, 30 years].

The participants of the focus group discussion stressed that at least two SBAs are required to provide continuous service delivery.

#### Inadequate equipment

**Fig 1:**
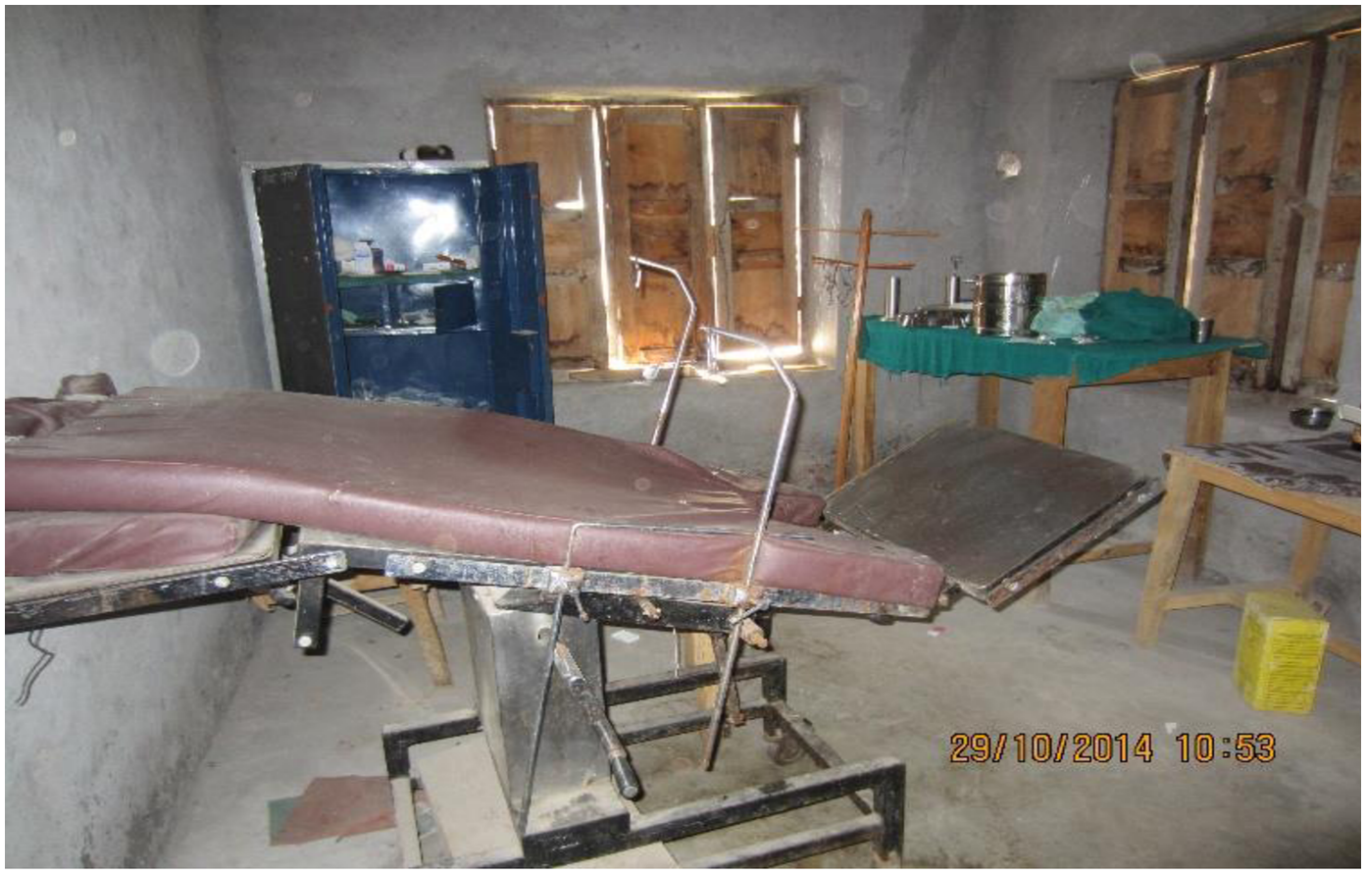
Labor room of a rural birthing center, Rukum, Nepal.

Service providers perceived that there was a lack of equipment and resources to enable them to provide a quality service. In addition, there was a lack of baby wrappers, and changing clothes for mothers. The infrastructure was also limited with a lack of basic facilities such as lighting, heating system, regular water supply and a placenta pit. The floors were cold, and the birthing center had only one old delivery bed that was broken (Fig 1).

The birthing center had two small rooms; one for waiting and the other for labor and giving birth. The single delivery room was not appropriate to provide skilled care at birth. Women during and post-labor require immediate support and attention, but it was reported that women often had to stay in a congested waiting room. Lack of space and privacy was a problem both for women and the SBA. A woman told her what she experienced:

“I saw another woman in labor at the same time I was on the delivery bed. She had given birth to her baby on the cold floor of the waiting room”

*[A woman who had given birth at the birthing center, 29 years]*.

Inadequate equipment and a lack of proper utilization of available resources meant that women had to face challenges. One community leader said:

“The center has inadequate equipments for childbirth or space for labor room. The solar system was installed for lighting, but it is not functioning”

*[A community leader, 30 years]*.

#### Poor quality of services

The perception of poor quality of care was the product of inadequate equipment and human resources and having an unskilled SBA for the management of complicated cases. Most (nine of the ten) women who had given birth perceived that there was no homely environment for mothers and newborns at the birthing centre. They lacked even the basic supplies during labor or post-birth such as warm clothes, water and food which diminished the quality of services. One service user shared her feelings:

“Sometimes I feel that institutional childbirth service is worse than that at home. At home, women get warm environment and food to eat. However, here [at the birthing centre] we have to wait for a long time for the service providers to arrive before getting any service and nothing is available”

[Woman, 25 years, who gave birth at the birthing center].

Service providers also perceived that the service provided through the birthing center did not enable quality care because of the lack of basic facilities. They were not able to meet the standards set in their guidelines. For instance, the infection control prevention was poor, the recent guidelines on labor and birth care were lacking, and the SBA was not updated (for example, she had forgotten the basic procedures to handle common obstetric emergencies).

Participants in the focus group discussion perceived that there was no difference between giving birth at home or at the birthing center. If complications arose in the birthing center, the SBA would not be able to manage them and would need to refer the woman. One local leader said:

“We need good care when complications arise. When we are sure that such cases will be referred, why should we go there? It would be four hours quicker to reach district hospital instead of going there”

*[Chairperson of ward citizen forum, ward number nine, 37 years]*.

One member of the management committee saw the birthing center during his visit and shared his feelings about the state of cleanliness of the birthing center:

“The birthing center was not in a clean condition; the floor was littered with soil, dust and pieces of paper. It was unmanaged, had a dirty toilet, poor storage and office facilities, and it had no water supply system”

*[Male, 39 years]*.

#### Poor governance (coordination and support)

Poor coordination and support between providers, local community and district health office was also found to influence the services offered by the birthing center. For example, the distribution of the maternity incentive cash was only made to women three months after they had given birth at the birthing centre. The women expected this payment much sooner. The reason cited for the delay was due to the delayed release of budget from the district health office. In addition, there was also a delay in the supply of essential equipment and drugs. Service providers, especially those working in the remotest areas like this study village, perceived that they were often overlooked to participate in training for skill enhancement, were less mentored and their work was not well recognized. They felt the need for non-monetary motivational training opportunities or exposure to other sites to enhance their skills in managing women with complications.

Women also had less trust in the financial scheme because it was minimal and the delay in receiving the cash incentive was almost like not having an incentive at all. In the case of delayed disbursement, it was also usually handed to the husbands who often spent it on themselves. One service user said:

“I did not get any money at the birthing center after childbirth, but my husband later told me that he received NRs. 1000 (≈10 USD) from the health worker after four months. However, I have no idea where he spent the money”

*[Woman who had given birth at the birthing center, 36 years]*.

Service providers did not receive as much support as expected from the community. This includes support for safety and security such as fencing, construction of the pathway to the birthing center, and local resource mobilization for hiring the SBA.

### Contextual barriers

#### Difficult geography

The geography and terrain were significant barriers to care seeking and utiliszation and were raised by almost all study participants [Table 2]. The study area itself was the remotest part of the district. Most wards were far away from the birthing center with many streams and hills on the way. Having had no roadway connection meant that it took at least 8 hours to reach the nearest motorway.

Nine in ten women felt that the birthing center was far away and very difficult to access. They had no means of transportation. The only option to carry a pregnant woman to the birthing center was a human-made stretcher. Given such conditions, going to the birthing center was even harder when the labor started late at night. A woman who lived far from the birthing center (ward number nine), said:

“The birthing center is in a very inaccessible location. We have to cross big hills, walk up and down and pass a few streams on the way to the center. During the rainy season, it becomes more difficult because there are no bridges over the stream. For us, it is better to give birth at home rather than risk being stranded on the way”

*[A woman who had given birth at home, 20 years]*.

The man whose wife had died in childbirth due to maternal causes perceived that physical distance with no road access for transportation was the primary reason behind the death of his wife. He shared his story as:

“I married Indira (name changed) (18), a girl belonging to the upper caste, after falling in love, without family consent. She became untouchable because of caste-based discrimination and was not allowed to go to her parent’s home. We faced many challenges to integrating into the society, and it was very hard for us. We were guilty in the society because inter-caste marriage was against the societal norm. After few months, when she became pregnant, we were not able to make the birth preparedness plan. She did not make ANC visits. When Indira went into labor late at night, she had a prolonged labor. At midnight, it would be a difficult journey to the birthing center due to an uphill walk. Next day late in the morning when Indira’s condition became worse, we decided to take her to the birthing center. We finally reached there the next day at noon. Indira was bleeding which is a potentially life-threatening condition. The SBA informed us that she had breach pregnancy which was not manageable at their level and they referred her to the district hospital. The referral center was not near (requiring two days to reach). After 8 hours of travel, when we reached motorway Indira died of haemorrhage”

[A husband, Dalit, 20 years].

**Fig 2:**
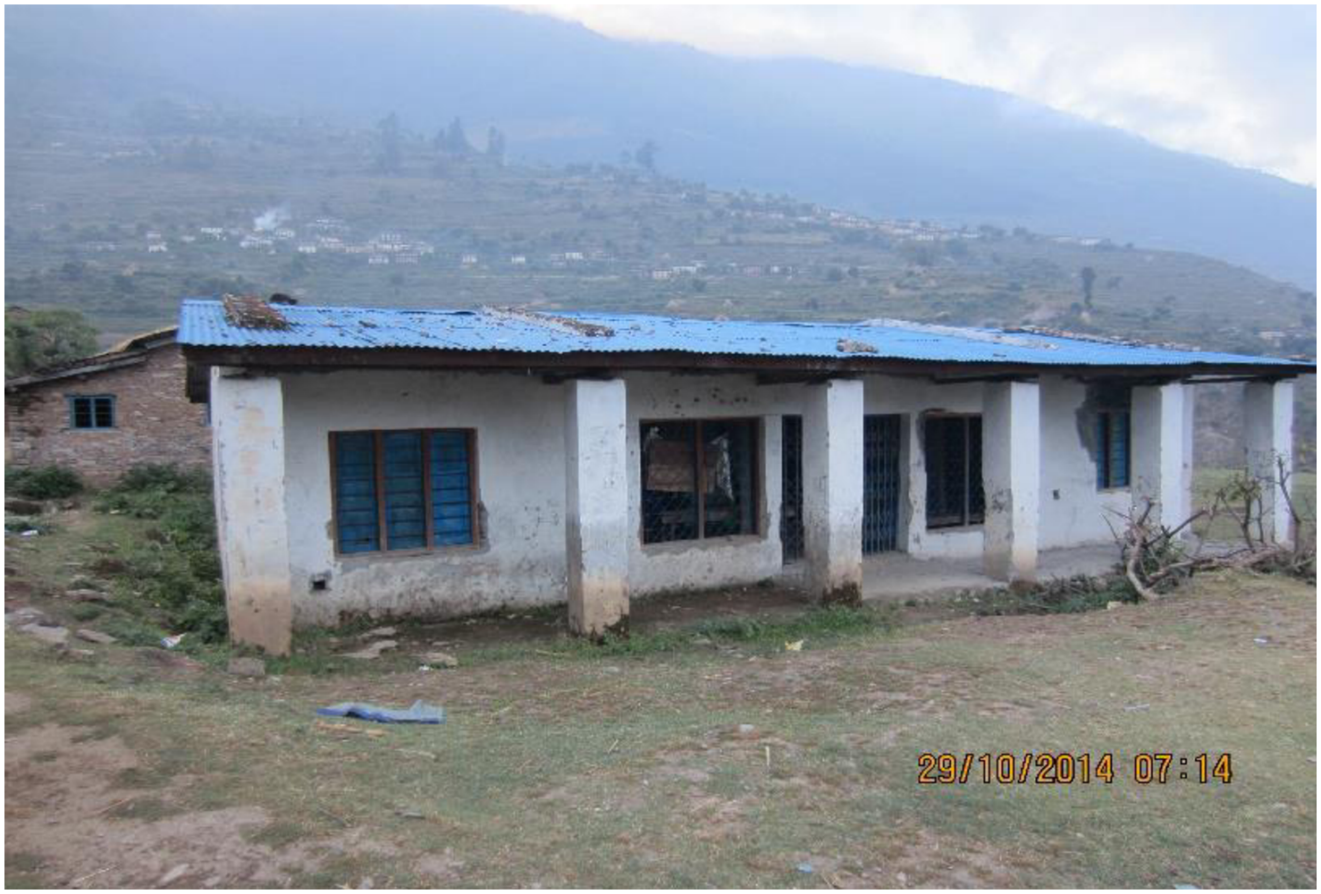
Building of a birthing center and community, Rukum, Nepal.

Most of the focus group participants including service providers reported that the birthing center was established at the current location after lengthy discussions between local leaders of the political parties. This place was chosen when local leader of the nearby ward was in power. There was inadequate community consultation when selecting the site for the birthing center. That was the reason why it was very inaccessible for some wards. For the largest ward (number nine), it was easier and quicker to reach the motorway to go directly to the referral center instead of going to the birthing center (Fig 2).

#### Poor birth preparedness plan and practices

Women explained that they were often engaged in household chores and taking care of other children and thus had no time to attend the birthing center for antenatal care (ANC) visits. This meant that many women who gave birth at home had inadequate knowledge on the dangers signs in pregnancy or any possible complications. The women who visited birthing center reported they were also not adequately counseled. One woman said:

“Didi (Sister in English, here referring to SBA) gave me some iron tablets and a deworming tablet. There was a long queue of service users, and all of them wanted to go back home faster. So, she had no time to explain to everyone in detail”

*[Pregnant woman, 28 years]*.

Most women (seven of ten) who had given birth at the birthing center had attended only after a prolonged labor. In most cases, the decision-making process for the facility-based birth was made by family members, especially mothers-in-law rather than the pregnant women themselves.

Regarding the plan for the place of birth, six of the ten women who had given birth at home said that they preferred to give birth at home as they felt it was most convenient. Furthermore, they believed that childbirth would be a natural process that did not require any special attention. They did not find the facility-based birth to be necessary because their previous births had occurred at home. However, if complications arose, they were prepared to visit the birthing center. One woman who gave birth at home said:

“Although, for me, home is very convenient for childbirth, if my life is in danger suddenly during childbirth, of course, I should be taken to the birthing center”

*[A woman who gave birth at home, 34 years]*.

Women who gave birth at home or in the birthing center had knowledge gaps about the maternity incentive program. The women had some knowledge about incentive for the institutional birth but little knowledge about the incentive for four ANC visits or about the amount of money. One woman who had given birth at the birthing center said:

“I know from the Female Community Health Volunteers that I will get some money if I give birth at the birthing center but I did not have an idea that one also gets money for making four ANC visits”

*[A woman who gave birth at the birthing center, 24 years]*.

Eight of the 10 women who gave birth at the birthing center who were aware and received the incentive said that the monetary incentive was minimal. One woman said:

“The transportation money given for institutional delivery is very minimal- nothing could be purchased from that small amount of money”

*[Woman who gave birth at the birthing center, 23 years]*.

Health workers also believed that women had limited birth preparedness practices. Even those who visited the birthing center wanted some physical materials to take home rather than just attending the counseling sessions.

#### Harmful local tradition and practices

Most women who gave birth at home were assisted by their mothers-in-law or senior women in the community. These untrained traditional birth attendants believed that they had given birth at home and assisted many such births, so it should not be a problem for other women to give birth at home. They were influential in the community. They also often sought care from traditional healers in the case of prolonged labor first rather than going directly to the birthing center. These practices further delayed timely care-seeking and put the women and newborn more at risk.

The focus group discussion participants also identified that they practiced specific cultural practices. For instance, if a woman had a prolonged labor, then metallic objects (such as local equipment made from iron), were touched on the woman’s abdomen. People believed that this made the labor pain pass away faster. When women had a retained placenta, some sort of heavy material was usually tied on the cord to force it down and facilitate the expulsion.

Health workers also shared their own experiences of health seeking behaviors of women. Sometimes even family members (senior members such as fathers-in-law, male, priests) refused to touch women in labor pain or post-birth due to the belief that God becomes angry if they did so. Dalit (so-called lower caste) women were rarely touched by people belonging to the so-called higher caste. The SBA shared a story:

“A Dalit woman gave birth to a baby at the birthing center six months ago. She experienced some pain post-delivery and felt uncomfortable. Then I requested an old woman to go nearby and look at the newborn, but she refused. Later I found out that she belonged to the Brahmin caste and was a wife of a priest”

*[SBA, birthing center]*.

#### Low level of trust

Women and community key informants had a low level of trust in the birthing center because of various reasons including previous poor experiences. They believed that it would be better not to attend the birthing center as the services were neither regular nor adequate. More importantly, they believed that the SBA could not manage women with complexities or complications. In short, there was a lack of clarity among the community people with regards to their expectations and the actual service delivery possible at the level of a birthing center which had limited physical and human services.

Even the community key participants seemed to trust the local pharmacy worker more than the SBA. They said that, at times, this worker was requested to assist with births at home, rather than the health workers working at the birthing center as he was more available. Community members did not know whether he was adequately skilled in handling childbirth but he was trusted during difficult times.

## Discussion

This study explored nine barriers to the utilization of a rural birthing center. Systematic barriers included the irregular and unreliable services, poor quality of services and inadequate resources. The contextual barriers included difficult geography and harmful cultural practices.

Health system barriers had affected the birthing center services and were intersecting and interdependent. For instance, the reasons for irregular and poor quality services were also due to inadequate equipment, understaffing or a poorly skilled provider and limited support and management. Women in labor seemed reluctant to attend the birthing center. Even if they reached the center, they had to wait for service providers, and they were further dissatisfied with the unfriendly environment. Women and the community perceived that the SBA was unable to effectively manage complications leading to a poor quality of service. Poor quality of services and referral have also been reported as the reason why women preferred giving birth at home in studies conducted in other countries such as Ethiopia and Bangladesh [38, 39]. Inadequate staffing and absenteeism have been reported as barriers to service availability in a previous study undertaken in Palpa, Nepal [40]. Similarly, studies conducted in Kaski and Chitwan in Nepal [41]also reported that poor quality of services was the reason for women to bypass the local birthing center while better facilities were available within an accessible distance [27]. However, in our study, options of bypassing were not readily available, so women gave birth either at home, or they chose to use the birthing center as the last resort if complications arose. The poor management and lack of coordination between different stakeholders further compounded the existing health system challenges and led to poor governance which ultimately resulted in substandard services [42]. Our study also identified governance challenges including short opening hours, poor supplies, human resources and community support. Community-based planning and management are quite successful in solving system challenges in a resource-poor setting in Africa [43–45], and this could be a lesson for the Nepalese context.

Contextual, that is, social or geographic, barriers also contributed to the poor utilization of health services. In our study, difficult geography was the main reason for poor access to the birthing center. The terrain and long walking hours for both women and services providers are well-known barriers to accessing health services in Nepal [46]. The time taken to reach the health institution and the waiting time affect the income of women indirectly (for example through lost daily wages), which had also been observed in a study in South East Nigeria [47] and Indonesia [48]. In our study, women were not able to get sufficient information on pregnancy care or information on incentives and services. All these factors meant that there was a low level of trust on available services and health service providers. These might be additional reasons behind the low use of birthing center as past studies have reported that birth preparedness and complication readiness were effective strategies for promotion of institutional births [49, 50].

Our study provides a unique example of how rural health institutions function and the services that are provided to the communities. Our findings are likely to be broadly similar to other inaccessible settings in the hills and mountains of Nepal [20]. In the hill and mountain regions of Nepal, birthing centers services are affected more by both contextual and systematic barriers. Women in these communities often have no option and, therefore, give birth at home. In the southern plains (Terai)[41]and where better transportation is available, women often go directly to the larger hospitals despite overcrowding being frequently observed [25, 51].

Some of the findings of our study are different from other studies. Firstly, unlike other studies which reported only the barriers identified by users or providers or both [20, 52], this study also included the responses of community key informants and we were able to triangulate the level of the barriers among all participants [Table 3]. Secondly, unlike the findings in other studies [20, 36, 52], women in our study did not prioritize economic barriers as the barriers to the utilization of birthing center. Instead, their focus was on the availability of service providers and services when they were in need. In the Maternity Incentive Scheme, incentives are provided to women to address the demand side financial barriers [23]and to encourage women to give birth at birthing centers, which is not a guarantee of the availability of standard care in the rural settings. Strengthening and further promotion of the Maternity Incentive Scheme would ensure not only incentives and promotion of SBA services but also the provision of quality services from birthing centers. Finally, unlike other studies which reported disparities among different social groups, we noticed that similar proportion of women had used birthing services (with the exception of Dalits- the so-called low caste groups).

Our study has highlighted possible strategies that might have relevance, and could enhance quality service delivery if such interventions are implemented at different levels. At the individual and household level, various promotional and health communication activities via mass media including local radio programs could be useful [53, 54]. Home visits by service providers and Female Community Health Volunteers to pregnant women, individual counseling, recording of every pregnant woman and follow-up of her pregnancy and delivery may also be important in the provision of quality care and ensuring a positive pregnancy outcome [55].

Similarly, at the community level, female community health volunteers can conduct counseling/health education sessions on birth preparedness plan at the monthly mothers' group meetings [55, 56]. Awareness raising activities could be conducted through mobilization of community social groups, community-based organizations, and users’ groups [57]. Health campaigns and street dramas are useful against harmful cultural practices and reduce the stigma associated with it [58], which in turn could increase the utilization of the birthing center.

At the birthing center level, different time and resource specific strategies may be adopted. Community-based planning and management might be an effective tool to identify problems, prioritization and local resource mobilization to problem-solving at the local level [43, 45]. This strategy might be helpful in making birthing center homely for women, providing warm clothes, improving cleanliness, heating and ensuring a water supply. Short-term works to address health system barriers could be improved with timely supplies of medicine, incentives and equipment [59]. Skill enhancement of the SBA, such as periodic mentoring/training and other non-monetary incentives [59], would be important to support. More effective means to hire SBAs and other staff members are important as is the provision of support for security and safety of the birthing center, including fencing and wall construction.

The long-term strategies that need resources and support from the higher level are construction works such as health facility building and road networks [60]. These include improving road access, or establishing a birthing center at a strategic location with proximity to a market which will attract more women. The concept of maternity waiting home, which has been tested in many countries [61, 62], might be a useful model where difficult geography and transportation system is poor. Other construction works would be the construction of accommodation or dormitory area for SBA close to the birthing center. Long term activities could be the inclusion of SBA training package into the auxiliary nurse midwife training, which will potentially minimize the acute shortage of SBA ensuring that all auxiliary nurse midwives graduated with the skills required in birthing centers [63].

Our study has some strengths and limitations. It was conducted among poor (and Dalit) women living in a remote part of the country using multiple data collection methods and participants. The importance of the common barriers was also qualitatively ranked according to respondent categories. The study was conducted in an inaccessible birthing center of a hill district, and thus the findings might not be generalizable to other accessible birthing centers of Terai (the Plain region) despite the quality and utilization of those centers being similar. Although service providers shared their external systematic problems, we could not capture the internal problems of the staff members. Moreover, Nepal has been devoid of locally elected government for the last 18 years, it was not possible to include elected executives of the local council, who are members of the birthingcenter management committee in the study.

### Conclusion

Our study has shown that this rural birthing centerwas not providing quality of care with limited service provision and poor trust from the community. Inadequate resources and poor governance were found to systematically hamper the service delivery. Difficult geographic terrain, harmful cultural practices, and poor birth preparedness plan were some of the reasons behind low level of trust in the birthing center services and low utilization of the services offered. Strategies for awareness campaigns, community mobilization, and various health system strengthening approaches could improve the utilizationof the rural birthing center.

## Supporting information

S1 Text File Questions guide for in-depth interviews and focus group discussions. (DOCX)
