# Supplementary material for: Barriers to utilization of childbirth services of a rural birthing center in Nepal: A qualitative study
Source: PLoS One. 2017 May 11;12(5):e0177602. doi: 10.1371/journal.pone.0177602 (PMC5426683; doi:10.1371/journal.pone.0177602)
Supplement: S1 Text File — (DOCX) [file pone.0177602.s001.docx]

# Question guides

**Socioeconomic and demographic characteristics**

- Name …………………….
- Place of residence (ward number) …………………
- Your age (years)…………………
- Sex: (a) Male (b) Female
- Education level/Years of schooling: (a) Illiterate (no schooling) (b) Primary (1-8 years) (c) Secondary (9-12 years) (d) Higher education (12+ years)
- Occupation: (a) Agriculture (b) Business (c) Social services(d) Job- Employee (e)Housewife
- Caste/Ethnicity: (a) Brahmin (b) Chhetri (c) Indigenous (Magar) (d.) Dalit (e) Other
- Religion: (a) Hindu (b) Buddhist (c) Christian (d) Other
- Marital status: (a) Married (b) Unmarried (c) Widowed (d) Divorced
- Food sufficiency through own farm produce: (a) Less than 3 months (b) 3 to 6 months (c) 6 to 9 months (d) Throughout the year
- Socioeconomic status: (a) Poor (food sufficiency less than year-round on own farm produce) (b) Middle class (Food security ensured throughout the year)

**Questions guide to service users (in-depth interviews)**

- Did you receive antenatal care services/childbirth services from the birthing centre during pregnancy? What did you do in your last births? Do you know the meaning of birth preparedness plan?
- Where did you give births? and why?
- What are the problems that women face at the birthing center ?
- Has birthing center been providing maternity care services whenever women are in need?
- Are childbirth services of the birthing center of good quality? Are you satisfied with the services/health workers? Why? Why not if no?
- What are the factors affecting provision of standard quality of facility-based births?
- Is the birthing center/skilled birth attendant competent enough to handle the complicated cases? If not, what do they do?
- Does birthing center have adequate resources to provide childbirth services?
- Why do women give birth at home instead of going to the birthing center? Who assist(s) such home-based births?
- How long does it take to reach the birthing center from your home? What means of transportation was used when you visited the last time?
- Do you know about the incentive program in your birthing center (the question to be explained in common language)? Is the allowance sufficient in terms of amount? Do you know about the maternity incentive program/transporation cost for facility delivery and four antenatal care visits?
- Do you have any beliefs that do not allow going to the birthing center?
- Are there any other reasons for non-use of birthing center services for childbirth practices? Please explain……………….

**Questions guide to health service providers (in-depth interviews)**

- What are the barriers towards the use of facility-based births
- What do you think about the quality of services provided through the birthing center? Is it up to standard?
- In your openion what factors hindering the standard quality of facility-based births ?
- Do you get adequate support from community/ district health office and management committee?
- Do you have adequate resouces- service providers, medicine, logistics and others?
- How maternity incentive program encouraging women to give birth at birthing center?
- What are the noticeable local factors that hinder the use of services from birthing center?
- Do you have any problems to provide services as per maternity care guideline? What are the challenges?
- Do women satisfied childbirth services available at the birthing center?
- At what state and stage mother attend at birthing center for childbirth? Do people request for assistance delivery at home instead coming birthing center?
- What problem are you facing to deliver childbirth services from the birthing center?
- Any memorable positive/negative event of child birth practices? If any please share….
- Are there any reasons of poor utilization of birthing center? Explain……

**Questions guide to community key informants/stakeholders (focus group discussions)**

- What do you think the reasons of underutilization of childbirth services of birthing center?
- Is birthing center providing regular and quality services to community? What might be the resons of poor quality serivces?
- Do birthing center have adequate resources- e.g. bed, equipment, rooms, health workers and others?
- Do people have any traditional beliefs/cultural practices regarding childbirths?
- Can you list the reasons why women do not attend birthing center for childbirths?
- What is/are major factor/s not attending for institutional delivery despite maternity incentive?
- Do mother/their relative consulting you for advice of place of delivery?
- Are there any reasons of poor utilization of birthing center? Explain……
